# Surgical aspects of pediatric abdominal pain in the era of COVID-19: clinical consideration and outcomes

**DOI:** 10.3389/fped.2024.1400638

**Published:** 2024-09-30

**Authors:** Nezar Abo-Halawa, Mohamed A. Negm, Mohamed Arafa, Mohamed Fathy

**Affiliations:** ^1^ Pediatric Surgery, Faculty of Medicine, South Valley University, Qena, Egypt; ^2^ Faculty of Medicine, South Valley University, Qena, Egypt; ^3^ Faculty of Medicine, Tanta University, Tanta, Egypt; ^4^ Faculty of Medicine, Minia University, Minia, Egypt

**Keywords:** acute abdominal pain, pediatric, COVID-19, appendicitis, multisystemic inflammatory syndrome in children (MIS-C)

## Abstract

**Background:**

Acute abdominal pain in pediatrics is a medical emergency that requires special attention. During COVID-19 pandemic, this disease presented in pediatric age by different presentations including abdominal presentations.The affected children are presented with abdominal pain, which may be caused by surgical causes or by the virus itself that necessitate surgical consultation.

**Purpose:**

This study highlights the impact of the coronavirus pandemic on pediatric patients with acute abdominal pain regarding the presentation, clinical evaluation, and surgical management.

**Methods:**

A retrospective cohort study was done through the collection of data from medical records and authors’ data repositories of pediatric patients presented with acute abdomen from March 2020 to March 2022, in three pediatric surgery tertiary centers.

**Results:**

Eighty-four pediatric patients with acute abdominal pain were included in this study. The diagnosis of acute appendicitis was found in 31 patients (36.9%). Generalized abdominal pain was noted in 17 patients (20.2%) and presentation mimicked acute cholecystitis was occured in 14 patients (16.7%). ultrasonography revealed intussusception in 12 cases (14.3%). Multisystem inflammatory syndrome in children (MIS-C) was present in 9 cases (10.7%) and only one case of pancreatitis (1.2%). Conservative management was successful in 66 cases (78.6%), while operative intervention was needed in18 cases (21.4%).

**Conclusion:**

During the COVID-19 pandemic, acute abdominal pain in children was frequently observed. Careful follow up is critically important as most cases do not necessitate surgical intervention. It is crucial to consider COVID-19 as a differential diagnosis in children presenting with acute abdominal pain, particularly in cases of atypical appendicitis and intussusception to prevent unnecessary surgical procedures.

## Introduction

Globally, only 1%–3% of documented COVID-19 cases occur in children. Nevertheless, children exhibit a higher propensity for gastrointestinal (GI) symptoms, with approximately 25% displaying at least one GI symptom. In some instances, GI symptoms may be the sole presenting symptoms of COVID-19 in children. The most reported GI symptoms are abdominal pain, diarrhea, and nausea/vomiting (1, 2).

A significant dilemma exists concerning the etiology of acute abdominal pain in children and the complications associated with multisystemic inflammatory syndrome in children (MIS-C). A diverse range of potential causes includes mesenteric adenitis, appendicitis, abdominal fluid collection, pancreatitis, terminal ileitis, cholecystitis, and intussusception (3–5).

Extensive research has been conducted on the gastrointestinal manifestations of COVID-19 in pediatric cases, including acute abdominal pain. However, currently, no study provides a quantitative analysis of abdominal pain cases from a surgical standpoint (4, 6, 7).

COVID-19 can induce abdominal pain resembling that in abdominal emergencies, particularly in MIS-C cases. These patients may exhibit symptoms akin to acute enteritis or an acute abdomen requiring surgery, posing a risk of misdiagnosis (8). According to AL Vecchio et al. (4), only 60% of children with COVID-19 who underwent surgery for acute appendicitis received a histologically confirmed diagnosis of acute appendicitis. Additionally, certain studies have indicated instances where children underwent emergency abdominal surgery for acute conditions only to have negative intraoperative diagnoses (9–12).

This study explored how the COVID-19 pandemic has affected pediatric patients’ presentation, assessment, and surgical management of acute abdominal pain.

## Patients and methods

### Study cohort

Following institutional review board approval, we conducted a retrospective study at three pediatric surgery tertiary centers in Egypt. All pediatric patients presenting with acute abdomen were included from March 2020 to March 2022. Data were collected from medical records and authors’ data repositories.

### Inclusion criteria

- Age <18 years
- Patients with abdominal pain and diagnosis of COVID-19 according to the protocol of diagnosis in our country (13).
- Patients with abdominal pain and diagnostic criteria of MIS-C according to the WHO definition and diagnosis of MIS-C (14, 15).

### Exclusion criteria

- Patients with incomplete data

### Patients’ presentation and data collection

- Demographic data
- Gastrointestinal symptoms, characteristics of abdominal pain
- Diagnosis of COVID-19 according to the protocol of our medical authority (13).
- Laboratory investigations and imaging studies
- Surgical management
- Hospital stay and follow-up

### Statistical analysis

The data were presented as numbers and percentages for qualitative data, while mean and standard deviation were used for quantitative data. Normality testing was performed using the Shapiro-Wilk test. Comparisons were conducted using the Mann-Whitney U test for non-normally distributed continuous variables and the independent *T*-test for normally distributed variables. Fisher's exact test was applied for categorical variables, with a significance level of *P* < 0.05.

## Results

### Clinical characteristics

During the review period, 223 children presented with GI manifestations of COVID-19. Ninety-eight (43.94%) of these children presented with acute abdominal pain. However, 14 cases were excluded due to incomplete data; therefore, 84 patients fulfilled the criteria of this study.

The age of the patients ranged from 6 months to 15 years (mean 8.51 ± 2.96 years). After performing ultrasound examinations (Appendix 1) and necessary laboratory investigations, the predominant presentation was right iliac fossa pain with a provisional diagnosis of acute appendicitis in 31 cases (36.9%). Generalized abdominal pain was noted in 17 cases (20.2%), ultrasonography revealed intussusception in 12 patients (14.3%), and 9 patients (10.7%) presented with MIS-C. The presentation mimicked acute cholecystitis was found in 14 patients (16.7%), and only one case presented with pancreatitis. There was no significant correlation between demographic data of the studied patients, likely due to the small sample size as shown in Table 1.

**Table 1:** Frequency of different possible diagnoses of acute abdomen.

Presenting symptoms	Total No of patients = 84	Age Mean ± SD	Male *N* = 48 (57%)	Female *N* = 36 (43%)	*P*-Value
Acute appendicitis (*N* = 31)	31 (36.9%)	10.6 ± 2.53	19 (61.3)	12 (38.7)	0.57
Diffuse abdominal pain (*N* = 17)	17 (20.2%)	7.41 ± 1.91	9 (52.9)	8 (47.1)	0.6
Cases mimic acute cholecystitis. (*N* = 14)	14 (16.7%)	8.79 ± 1.63	7 (50)	7 (50)	0.55
Intussusception (*N* = 12)	12 (14.3%)	4.83 ± 2.72	7 (58.3)	5 (41.7)	0.9
Multisystemic inflammatory syndrome in children (MIS-C) (*N* = 9)	9 (10.7%)	8.00 ± 2.35	5 (55.5)	4 (44.5)	
Pancreatitis (*N* = 1)	1 (1.2%)	8.00	1	0	–

### Surgical management

#### Acute appendicitis

Of the 31 patients presenting with acute appendicitis, surgical intervention was performed in 9 cases (8 laparotomies and 1 laparoscopy). Surgical findings in the first two cases revealed a non-inflamed appendix with congested serosa, enlarged mesenteric lymph nodes, and a considerable amount of peritoneal fluid. The remaining 7 cases were positive for purulent appendicitis.

#### Generalized abdominal pain

In patients presenting with generalized abdominal pain (*n* = 17), 5 cases (29.4%) required surgical intervention. Surgical findings in one of these cases included multiple perforations of the rectosigmoid and descending colon, diffuse wall thickening of the remaining colon, and an adherent pyogenic membrane, necessitating an ileostomy.

#### Intussusception

Out of 12 patients, 9 cases showed transient radiological intussusception with mild abdominal pain (7 cases presented with small bowel intussusception and 2 cases with ileocolic intussusception), all of which showed spontaneous resolution during follow-up after conservative treatment.

Hydrostatic reduction was performed in 2 cases with ileocolic intussusception, but it failed in one case, necessitating laparotomy. This revealed colo-colic intussusception and multiple enlarged mesenteric lymph nodes. One case with peritonitis and intestinal gangrene required bowel resection and anastomosis (Table 2).

**Table 2:** Comparison of different possible diagnoses of acute abdomen regarding line of management.

	Conservative	Type of Surgery			*P*-value
		Drainage	Laparotomy	Laparoscopy	
Acute appendicitis (*N* = 31)	22 (71%)	0	8	1	0.17
Diffuse abdominal pain (*N* = 17)	12 (70.6%)	2	2	1	
Cases mimic acute cholecystitis (*N* = 14)	14 (100%)	0	0	0	
Iintussusception (*N* = 12)	10 (83.3%)	0	2	0	
Multisystemic inflammatory syndrome in children (MIS-C) (*N* = 9)	7 (77.7%)	2	0	0	
Pancreatitis (*N* = 1)	1	0	0	0	

#### Operative vs. non-operative outcomes

Our findings revealed a notable shift in managing acute abdomen during COVID-19, with successful conservative treatment in 66 (78.6%) patients, while 18 (21.4%) underwent surgical management, including laparotomy, laparoscopy, or drainage. There was no statistically significant difference between the groups (*p* = 0.17), likely due to the small sample size. Hospital stays ranged from 8 to 29 days (mean: 16.92 ± 3.55 days). Age variation was insignificant between the groups with different presentations, as detailed in Tables 2, 3.

**Table 3:** Line of treatment according to age and gender.

	All cases (*N* = 84)	Conservative (*N* = 66)	Surgery (*N* = 18)	*P*-value
Age (mean ± SD)	8.51 ± 2.96	8.35 ± 3.03	9.11 ± 2.70	0.33
Age groups frequency:				0.78
Less than 2 years	1 (1.2)	1 (1.5)	0 (0)	
2–12 years old	76 (90.5)	60 (90.9)	16 (88.9)	
12–15 years	7 (8.3)	5 (7.6)	2 (11.1)	
Gender- *N* (%)				0.70
Male	48 (57.1)	37 (56.1)	11 (61.1)	
Female	36 (42.9)	29 (43.9)	7 (38.9)	

## Discussion

The coronavirus pandemic represents a medical paradigm shift, influencing our comprehension and treatment strategies for various illnesses. The decision to undergo surgical treatment can be problematic for children with COVID-19 due to the increased risk of postoperative complications. Therefore, it is generally advisable to avoid surgery unless deemed necessary (16, 17).

The current investigation identified 31 patients with appendicitis. The first case revealed intraoperative findings of a congested appendix with mesenteric adenitis and ileitis accompanied by peritoneal fluid. Unfortunately, this case experienced a challenging postoperative period, which caused the development of MIS-C. In the second case, diagnostic laparoscopy exhibited findings similar to the first but was managed conservatively. Subsequently, four complicated cases underwent appendectomy initially, while the remaining 25 cases received conservative treatment, with only three requiring subsequent surgery.

Overall, surgery was conducted in 29% of cases presenting with appendicitis. COVID-19 can lead to acute abdomen that mimics appendicitis, as supported by existing literature indicating cases where appendectomy was performed with histologically proven negative results (4, 6, 16). Conversely, during COVID-19, severe gastrointestinal involvement, including ileitis and mesenteric adenitis, can occasionally mimic appendicitis (3, 18, 19). Similar to our findings, there is a noticeable shift toward conservative treatment of appendicitis during the COVID-19 pandemic (20–24).

Another significant finding involves 17 cases presenting with generalized abdominal pain. The initial laparotomy in the first case revealed peritoneal fluid in the pelvis, right and left iliac fossa, hepatorenal pouch, mesenteric adenitis, and edematous intestine, necessitating drainage. The second case underwent laparoscopy, displaying diffuse edematous terminal ileum and colon with intra-abdominal fluid and mesenteric adenitis. Subsequently, two cases received drainage in the right iliac fossa, while the remaining 14 cases were treated conservatively with spontaneous resolution, except in one case where laparotomy revealed sigmoid colon perforation. In four cases, conservative treatment was employed, and anticoagulant therapy was administered due to suspicion of mesenteric thrombosis based on duplex study findings of mesenteric vessels.

One case showed multiple perforations in the rectosigmoid region and descending colon. The involvement of the sigmoid colon may be attributed to evidence suggesting that the vascular effects of COVID-19, such as ischemic colitis, tend to impact the left colonic flexure and sigmoid colon more than the distal rectum, concerning their dual blood supply (25, 26).

Conservative methods proved effective in treating diffuse abdominal pain. This aligns with other studies recommending conservative treatment with regular reassessment, as most cases naturally resolve without intervention. Moreover, multiple lines of evidence indicate that intraperitoneal fluid results from increased intestinal permeability, and bacteriological examinations yield negative results (4, 5, 27).

Among patients in our study, 14 cases presented with symptoms resembling acute cholecystitis, particularly abdominal pain in the right hypochondrium. Ultrasound examinations revealed mild hepatomegaly with gallbladder wall thickening and mesenteric adenitis in three cases, mild hepatomegaly with a small amount of free fluid in six cases, and gallbladder wall thickening and edema with mesenteric adenitis in five cases. All these cases responded to conservative treatment.

The pathogenesis of hepatobiliary involvement is attributed to mild sinusoidal lymphocytic infiltration and sinusoidal dilatation. These factors result in mild pain and manifest on ultrasound as mild hepatomegaly with various gall bladder-related changes (28, 29).

Previous literature has documented intussusception as a cause of abdominal pain during COVID-19. However, no documented evidence of transient intussusception associated with COVID-19 exists in the literature (4). Transient intussusception is a distinct phenomenon that may impact children (30–32). The pathogenesis of transient intussusception associated with COVID-19 is not clearly defined, but it may be attributed to pathological features such as mesenteric adenitis, bowel wall thickness, and edema associated with COVID-19. This pathogenic mechanism could contribute to the occurrence of intussusception, as observed in cases involving other intestinal viruses (4, 33).

MIS-C is a systemic hyperinflammatory syndrome secondary to COVID-19 infection; its diagnostic criteria include abdominal pain, diarrhea, vomiting, colitis, ileitis, abnormal liver function tests, and ascites (14, 15, 34). It impacts multiple organs and can lead to shock with heart dysfunction, with predominant involvement of the gastrointestinal tract observed in 70%–90% of cases. The gastrointestinal symptoms associated with MIS-C may mimic those of an acute abdomen (33, 35). In this study, 9 cases with MIS-C presented with diffuse abdominal pain and ultrasound findings of mesenteric adenitis and ileitis with fluid collection. Drainage was performed in 2 cases, while the remaining 7 cases responded well to conservative treatment. Similarly, Lo Vecchio et al. performed drainage in 5 out of 21 children (23.8%), with most fluid collections resolving over time and the fluid being bacteriologically sterile (4).

Abdominal pain in MIS-C can manifest in different presentations, such as pseudo-appendicitis pain and diffuse abdominal pain. The associated pathological changes in the abdomen are diverse and may include mesenteric adenitis, ileitis, and thickening of the bowel loop, with a predominant presence of fluid collection (36, 37). Appendicitis in MIS-C is typically associated with a patent lumen, serositis, and perivasculitis. Conservative treatment approaches are recommended for these cases (4, 38).

Acute abdominal pain in children has traditionally been evaluated surgically to exclude common causes such as appendicitis or perforated intestine. However, the COVID-19 pandemic has necessitated a shift in approach due to similarities between COVID-19 and other coronaviruses affecting gastrointestinal enterocytes and causing abdominal pain (39). The primary challenge now is distinguishing between surgical causes of acute abdominal pain and those related to COVID-19, emphasizing the need for precise diagnostic methods to prevent medical errors and unnecessary surgeries.

While laparoscopic surgery is considered the gold standard for appropriate cases of acute abdominal pain, our study utilized laparoscopy in only 2 cases (11.1%) out of 18 patients. Initially, there was concern about the safety of laparoscopy during the pandemic, with some studies suggesting an increased risk of virus particle aerosolization during CO_2_ insufflation (40, 41).

Despite the WHO declaring on May 5, 2023, that COVID-19 is no longer an international public health emergency, the disease remains prevalent. Therefore, it is essential to consider COVID-19 as a potential cause of abdominal pain in all cases.

Our study's relatively small number of cases can be attributed to several factors. COVID-19 presentation in pediatric patients is less common than in adults, and respiratory symptoms are more prevalent than abdominal ones. Additionally, some cases were transferred to other centers following local protocols. Despite these limitations, our retrospective study provides valuable data on a globally significant disease. It captures a diverse range of presentations of acute abdomen in pediatrics, highlighting the need for further research to enhance our understanding of this complex issue.

## Conclusion

Acute abdominal pain in children during COVID-19 is a common occurrence, often resolving without surgical intervention due to the self-limiting nature of most gastrointestinal manifestations. It is crucial to consider COVID-19 as a differential diagnosis in patients presenting with acute abdominal pain, particularly in cases of atypical appendicitis and intussusception. Meticulous evaluation and accurate diagnosis are essential to avoid unnecessary surgical interventions in children with persistent abdominal pain.

## Data Availability

The original contributions presented in the study are included in the article/supplementary materials, further inquiries can be directed to the corresponding author.

## Appendix 1

### Ultrasound findings

#### In all cases especially MIS-C

• Mesenteric fat inflammation
• Intestinal wall thickening
• Mesenteric adenopathy
• Peritoneal fluid collection

#### US finding in cases mimic acute appendicitis

Appendix diameter 6–7 mm

• Hypoechoic wall not echogenic.
• Mildly inflamed ilium.
• Not encysted or turbid.
• Few mesenteric LNs.

#### US finding in cases mimic cholecystitis

Thickening of gall bladder wall, hepatomegaly, decreased echogenicity of the liver.

#### US finding in cases of intussusception

• 3 cases with ileo-colic intussusception in epigastrium and mesenteric adenitis with thickened ileum and free fluid
• 7 cases with ileo-ileal intussusception and mesenteric adenitis with thickened ileum and free fluid

2 cases with ileo-colic intussusception in Rt lumbar and mesenteric adenitis with thickened ileum and free fluid.
